# Annotations

**Published:** 1888-01-21

**Authors:** 


### ANNOTATIONS.

**An Inquiry Answered.**

"MAY I call your attention," writes "A Country Matron," "to an article in the *Lancet* of December 31st, entitled, 'Medical Ethics'? This, taken in conjunction with a former leaderette some numbers back in the same paper, mentioning THE HOSPITAL by name, and written in a very unpleasant tone, can but lead the public to suppose that THE HOSPITAL is again referred to. If so, will it not be as well to take notice of the same, as otherwise 'this project' is said to have ended in well-merited failure, and will lead the public to think THE HOSPITAL has come to an end?" This is a sample of the inquiries we are now receiving, and for the sake of our correspondents we "take notice" generally of what has been said. The only thing, however, we need say, is that the circulation of THE HOSPITAL is now larger than it has ever been at any previous time, being over ten thousand weekly, as against less than a-fifth of that number a year ago. If this be "failure" on the part of a paper which has not yet been in existence eighteen months, what can a circulation of seven or eight thousand a-week be on the part of a paper which has been in full operation for nearly half-a-century? Clearly, then, even the *Lancet* cannot mean that THE HOSPITAL is a "failure." To what, therefore, can it refer? Is it to the *National Pension Fund?* But the *Pension Fund*, which has been in course of evolution for fifteen years, is now, by the princely generosity of four City merchants, an accomplished fact. There are still some details to be worked out in connexion with premium rates, etc., but these, it is expected, will be all in order within the next fortnight, and it is intended that the fund shall be thrown open to subscribers on February 1st. If £20,000, placed to the credit of the Pension Fund be a "failure," then by all means let us have nothing but "failures" to the end of time. So far as we know, these are the only concerns in which this paper has an immediate interest at the present moment; and therefore the only subjects to which we need refer. We are aware, of course, that the *Lancet* has been striving very hard to give us a paternal and very effectual benediction; and as its efforts have proved real "blessings," though by no means "in disguise," we can well afford to forgive that journal and its conductors, which we hereby express our readiness to do.

**May a "Layman" do Good?**

What is a "Layman"? It is a dreadfully large and comprehensive question, and opens up such vistas and labyrinths of ecclesiastical, metaphysical, medical, scientific, and an infinite variety of other subtle polemics, that we "give it up." But, though we frankly confess our inability to define a "Layman," we are anxious to answer the question at the head of this Annotation; for if a layman is not permitted to do good *because he is a layman*, it is clear that everybody outside the learned professions is in a preparatory condition for something very terrible by-and-by. It is objected to a certain person, whom we need not name, by certain other persons, whom also we need not specify, that he is not a doctor, but only a benighted official or member of the Stock Exchange; and that, therefore, it is an offence and an impertinence on his part to take upon himself to endeavour to improve the condition of nurses and other hospital officials. The offence is all the deeper because the particular line which his efforts have taken has been a *financial* line, and everybody knows that no member or official of the Stock Exchange can possibly know anything at all about finance. It is equally a matter of universal notoriety that every medical man is *ipso facto* a born, trained, and accomplished financier. If, therefore, a Pension Fund for Nurses and Hospital Officials was to be started and successfully carried out, the proper thing to do was to secure the services of a doctor—any doctor—the first you could catch in the street, and put him at the head of the movement. Happily for the National Pension Fund, it will not be wrecked for want of doctors on its board of management. What is still more comforting, if not absolutely a thing to be rejoiced over for ever and ever, it is even said that Mr. Burdett, the very head centre of the whole scheme, is a doctor really, though not nominally. It is known, of course, that he spent fifteen active years in hospital work as secretary and in other capacities. But the facts which we here quote from the *Medical Times* of February 28th, 1885, are not generally known, and we have pleasure in reproducing them for the general edification. The *Medical Times* says: "In our last issue it was stated that Mr. H. C. Burdett is not a medical man. This, we are informed, is only true 'in the letter,' Mr. Burdett having taken out the full curriculum at Guy's, and passed his 'First College,' but having, for private reasons, abstained from taking out his diploma." This quotation will greatly comfort all those over-anxious persons who thought the National Pension Fund was bound to be a failure for want of an accomplished financier in the shape of a medical man at its head. As showing the unique competence of Mr. Burdett for dealing with Hospital affairs generally, the following testimony from the *Lancet* is peculiarly appropriate at the present time: "Being anxious,' says the *Lancet*, "to help the London Hospitals at a time of great pecuniary depression like the present, we have resolved to try to raise the amount at the disposal of the Council of the Hospital Sunday to £40,000 before the distribution of the Fund on the 31st. *With this object we consulted Mr. Henry C. Burdett, whose interest in hospitals is well known, and who is widely recognised as the chief authority on the subject*" (the italics are ours) "and he kindly undertook to write the following historical sketch of the Hospital Sunday movement in the metropolis. *We have thought it desirable to obtain Mr. Burdett's permission to state that he is the author of this historical and helpful account of probably the most beneficent philanthropic movement of the time, as we believe the information will give force and weight to this interesting statement, whilst it will secure for it an attention from the benevolent and wealthy which it might otherwise fail to obtain*" (the italics are ours). *Lancet*, July 25, 1885, p. 174.

**The Charity Dispensing Society.**

Some people are always ready either to bless or to ban a new institution at a moment's notice. It is sometimes wise to do neither. There is a third course open to those who have a little judgment and patience; and that is, to wait and see what kind of work the institution shows itself able and willing to perform. It is not a new principle, or one devoid of high sanction that tells us a "tree is known by its fruit." A "Charity Dispensing Society" has been founded; and, as it would appear, mainly by persons who are dissatisfied with the Charity Organization Society. Already certain newspapers, more notorious for loud than for wise talk, are ruffling their feathers and craning their necks for a defiant crow. That is, to say the least, a little premature. We can all afford to wait for the fruit season. One circumstance appertaining to the new society is that it proposes to do its dispensing by honorary almoners. That is quite as it should be; and we may hope it will never depart in practice from so sound a principle. It appears that the new association is already regarded by some as the natural opponent of the Charity Organisation Society. Perhaps it is; but what then? The Charity Organisation Society will not collapse on that account. Everyone must admit the need there was for general and thorough inquiry when that society was founded; and the need which existed then is as great now, and is likely to continue as great as it ever was.

